# A New Tactic for Label-Free Recognition of β-Trophin via Electrochemiluminescent Signalling on an AuNPs Supported Immuno-Interface

**DOI:** 10.1038/s41598-017-11750-8

**Published:** 2017-09-11

**Authors:** Lijuan Zheng, Chen Fang, Jilin Yan, Huiling Li, Yifeng Tu

**Affiliations:** 1 Institute of Analytical Chemistry, Dushu Lake Campus, Soochow University, Industrial Park, Suzhou, 215123 P.R. China; 2 Second Affiliated Hospital, Soochow University, Suzhou, 215004 P.R. China; 3 College of Nursing, First Affiliated Hospital, Soochow University, Suzhou, 215006 P.R. China

## Abstract

In this paper, a new strategy is reported for preparing a label-free β-trophin electrochemiluminescent (ECL) immunosensor with good specificity, reproducibility and stability. An aquagel polymer from the hydrolysis of (3-aminopropyl) trimethoxysilane acted as the linker to catch the Au nanoparticles (AuNPs) on the indium-tin oxide (ITO) substrate by a two-step method. The AuNPs play an important role in enhancing ECL and immobilizing the β-trophin antibody. This immunosensor can test for β-trophin using luminol as an ECL probe. The ECL intensity at the resultant sensor, after the direct immuno-interaction, was proportional to the concentration of β-trophin and had a low limit of quantification as 4.2 ng mL^−1^. After deep discussions on the ECL mechanism of this immunosensor, we found that its sensitivity is greatly affected by the presence of oxygen and improved under deoxygenation. We believe that this sensor can be used for clinical cases.

## Introduction

β-Trophin (also known as hepatocellular carcinoma-associated protein-TD26, RIFL, angiopoietin-like protein 8, lipasin, and C19orf80) is a 198-amino acid protein (M_W_ = 22 kDa) that is overexpressed in liver or adipose tissue^1^ and was first included in GenBank in 2004 (https://www.ncbi.nlm.nih.gov/genbank/). By 2012, studies had shown that β-trophin might be involved in the metabolism of glucose^2^ or lipids^3–5^. In 2013, Melton *et al*. found that this peptide hormone can control the proliferation of pancreatic β cells in mice. Subsequent studies have indicated that β-trophin in the human body is closely related to diabetes^6^. These findings raised the hope for the development of novel therapeutic approaches with β-trophin as a drug target of diabetes or cardiovascular disease. However, these findings have been disputed by other authors since 2014, ultimately resulting in the retraction of the original paper in 2016^7–10^. Thus, the function of β-trophin in diabetes is once again uncertain. Meanwhile, there have been several severely conflicting reports about the β-trophin content from the pg mL^−1^ to μg mL^−1^ level in real serum samples^11–13^. Considering this dubious situation, we believe it is beneficial to more precisely quantify β-trophin content in related organismic tissues. To date, enzyme-linked immunosorbent assay (ELISA) is the most common technique for monitoring the content of β-trophin^1–9^. However, the application of ELISA in point-of-care testing (POCT) is limited by the high cost of ELISA kit and the bulky instrument^14^.

Different biosensors have been maturely developed for the diagnosis of known or unheard diseases by gauging biomarkers including proteins^14–17^. Immunoassays, including immunosensors, based on specific immune recognition have attracted growing attention in related fields, including medical diagnostics^18, 19^. There are many signalling channels, such as radioisotopes^20, 21^, ELISA^22, 23^, fluorescence^24^, electrochemistry^25^, piezoelectricity^26^, chemiluminescence and electrochemiluminescence (ECL), have been used for quantitative calibration. Among them, ECL is one of the most attractive techniques and has been applied extensively in pharmacology^27^, clinical chemistry^28^, and the analysis of food and water^29, 30^ due to its comprehensive advantages of high sensitivity, spatio-temporal controllability, low background noise and simplified setup^31–34^. The immunoassay is often a heterogeneous process with direct, competitive^35^ or sandwich^36^ modes. In contrast to the latter two modes, the direct immunoassay is relatively simple and fast, more suitable for real-time monitoring, and avoids time-consuming, laborious and high-cost antibody labelling. Furthermore, the label-free direct immunoassay will further reduce the labelling process, saving time and cost and reducing the risk of damaging the activities of the biomolecules during the chemical conjugation. Further promotion of the sensing ability and applicability of label-free direct ECL immunosensors has become a focus of research, and is a great challenge to perfectly meet the demands of POCT in clinical investigations.

Biocompatible nanomaterials are the best substrate for constructing successful biosensors. They are also inducted into the studies of ECL analysis and ECL biosensors to improve the quality of their analytical merits^37–42^. These studies have made significant progress not only in intensifying ECL emissions but also in reducing the requirements for high medium alkalinity and exciting potential. Thus, these nanomaterials provide an opportunity for the application of ECL in the physiological domain and for implementation as bio-detectors. Among them, metal nanoparticles, especially Au nanoparticles (AuNPs), have received most consideration because of their unique optical performance, appealing catalytic activity, good electrical conductivity and biocompatibility^43–45^.

In recent years, we have dedicated our efforts to developing ECL biosensors for diabetes-related indexes such as glucose^42^, glycosylated haemoglobin^46^, and genes^47^. It has already been demonstrated that the platform of ECL biosensing via an immune strategy on nanomaterials functionalized indium tin oxide (ITO) glass was competent for those purposes. Therefore, it is possible to build a label-free direct ECL immunosensor for β-trophin detection via the specific recognition of the β-trophin antibody.

## Results

### The investigation of immunosensor construction and its electrochemical behaviour

**Figure 1:**
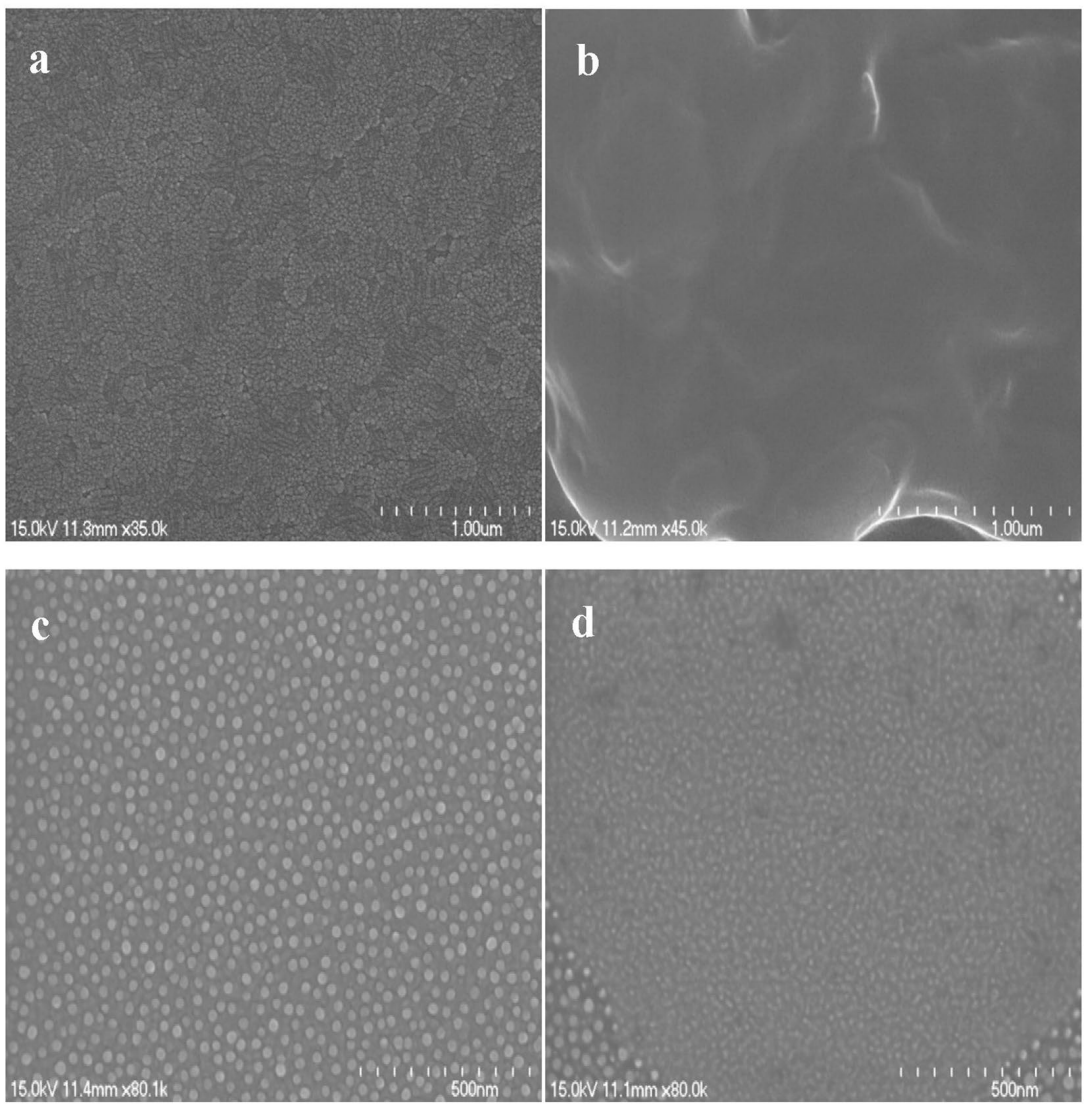
The SEM images of (**a**) bare ITO, (**b**) APTMS/ITO, (**c**) AuNPs/APTMS/ITO and (**d**) anti-β-trophin /AuNPs/APTMS/ITO.

An investigation with electron microscopy is direct and helpful for judging the sensing matrix. The changes in surface morphology of an electrode along with the sensor-preparing process are clearly displayed in Fig. 1. Picture “a” shows the surface of the ITO substrate. Then, it is covered by a cloudy film when the hydrolysed (3-aminopropyl) trimethoxysilane (APTMS) is overlaid (Fig. 1b). In picture “c” in Fig. 1, the SEM image of the AuNPs layer shows that AuNPs with a diameter of ~15 nm are arranged in an orderly fashion on the surface of the ITO through the adherence of APTMS, forming a mono-dispersed homogeneous decorating layer. This indicates that the hydrolysed APTMS can effectively place AuNPs onto the ITO substrate with excellent dispersion. Presumably, the AuNPs adhere to the substrate because of the electrostatic force between the negatively charged AuNPs and the positively charged amine groups of the hydrolysed APTMS. Figure 1d presents the morphology of the resultant immunosensing interface (anti-β-trophin/AuNPs/ITO). It is readily apparent that the anti-β-trophin proteins are bound tightly on the AuNPs/ITO, similar to a layer of paste. This indicates the successful conglutination of proteins on AuNPs.

**Figure 2:**
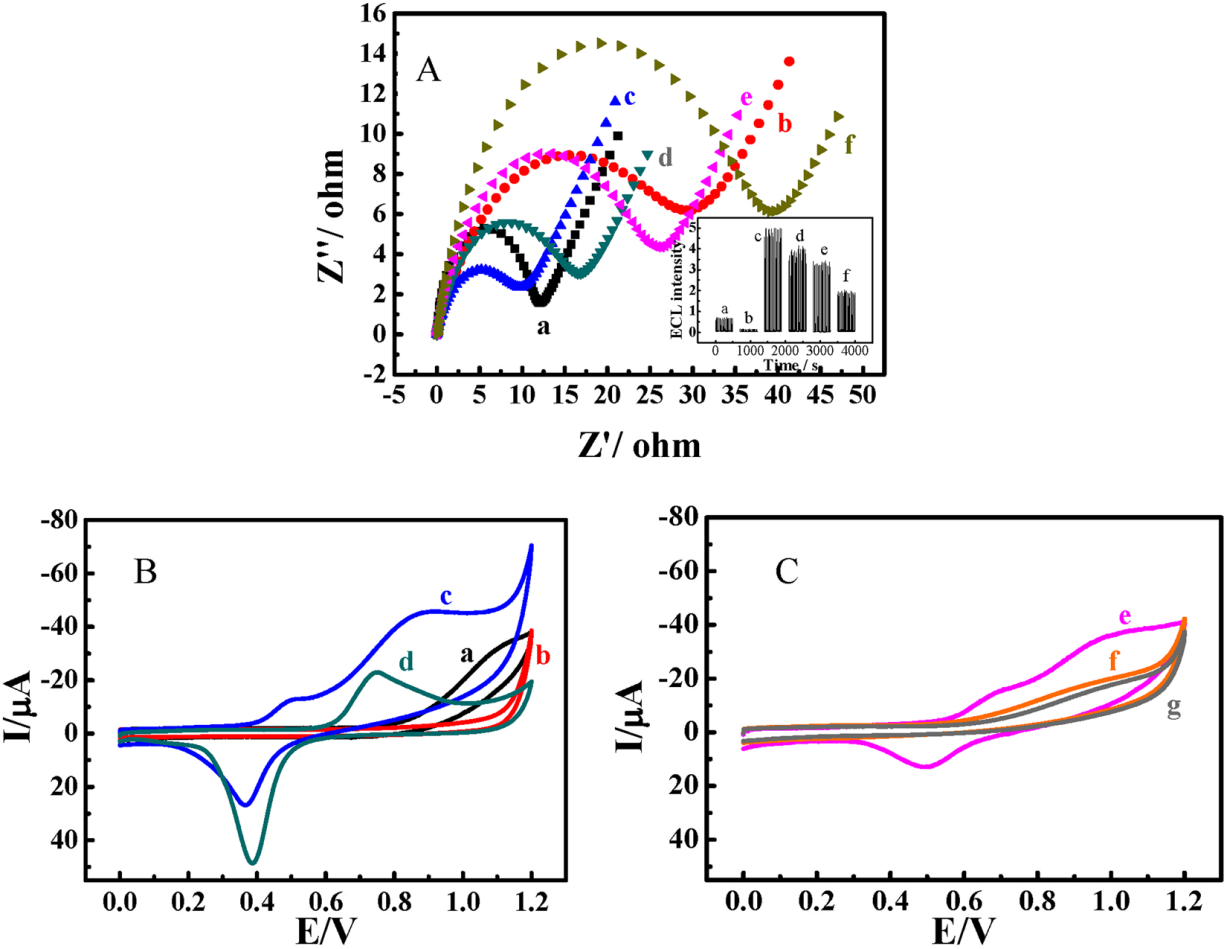
(**A**) The EIS of (a) bare ITO, (b) APTMS/ITO, (c) AuNPs/APTMS/ITO, (d) anti-β-trophin/AuNPs/APTMS/ITO, (e) BSA/anti-β-trophin/AuNPs/APTMS/ITO and (f) responded toward β-trophin in 0.1 mol·L^−1^ NaCl supporting electrolyte solution containing 5 mmol·L^−1^ [Fe(CN)_6_]^3−/4−^. The insert is the ECL signals of different surface status (pH 8.0, 5 × 10^−8^ mol·L^−1^ luminol); (**B**) The CVs of 0.1 m mol·L^−1^ luminol on (a) bare ITO, (b) APTMS/ITO, (c) AuNPs/APTMS/ITO and (d) bulk Au electrode in PBS (pH = 8.0) with a scan rate of 0.05 V·s;^−1^ (**C**) The CVs of 0.1 m mol·L^−1^ luminol on (e) anti-β-trophin/AuNPs/APTMS/ITO, (f) BSA/anti-β-trophin/AuNPs/APTMS/ITO and (g) responded toward β-trophin in PBS (pH = 8.0) with a scan rate of 0.05 V·s^−1^.

Electrochemical impedance spectroscopy (EIS) is also an effective technique for probing the surface character of an electrode, which is shown in Fig. 2A. The double layer capacitance or constant phase element (CPE, a non-intuitive capacitance other than double layer) and the electron transfer resistance (R_et_) will change with each step of the modification. As shown in Fig. 2A, compared to the ITO substrate (curve a), the electron transfer resistance (R_et_) increased obviously after non-conducting hydrolysed APTMS was attached to it (curve b); on the other hand, the AuNPs greatly accelerate the electron transference, as clearly seen in (c) of Fig. 2A. Meanwhile, the proteins of the antibody, BSA and antigen could resist the arrival of electrons to the sensing interface, leading to the increasing impedance (curves d, e and f), which proves the immobilization of those biomolecules. In correlation, the ECL signals of each step are displayed in the inset of Fig. 2A. These two pictures are consistent with each other, which demonstrates the attainment of our immunosensor.

Figure 2B displays the CV curves of 0.1 mM luminol on different electrodes in a buffer solution. Compared to bare ITO (curve a) or APTMS/ITO (curve b), there is almost no obvious Faradaic current of luminol, the redox peak currents are highly increased on the AuNPs decorated ITO (curve c). Here, two remarkable oxidation peaks at 0.537 V and 0.902 V and a reduced peak at 0.366 V are observed. This is in rough accordance with the redox behaviour of luminol on a bulk Au electrode (curve d) but at a lower potential. This result indicates that the AuNPs-functionalized electrode performs better than the bulk Au electrode for the oxidation of luminol due to the rising d-band electrons in AuNPs. The decreased voltage for the redox of luminol also indicates that the catalytic activity of AuNPs reduces the activation energy of the ECL reaction, thus accelerating the ECL reaction. With successive deposition of different proteins (antibody, BSA and antigen) on the electrode, the peak current gradually decreases (curves e, f and g in Fig. 2C), which also corresponds to the EIS and ECL tests.

### The optimization of preparation/detection conditions of immunosensor

There are many conditions that will affect the final performance of AuNPs functionalized ITO electrodes. Considering the possible interaction between these factors, an orthogonal optimization of L_16_(4^3^) was conducted, with the ECL emission of luminol on the resultant electrode as the target index. Table 1 shows the parameters and outputs of the orthogonal experiment with 4 levels of 3 factors.

**Table 1:** Process parameters and the results of L_16_(4^3^) orthogonal experiments.

No.	APTMs (v/v)	T (h)	Au (m/10^−3^ g/mL)	ECL intensity (V)
1	0.01%	0.5	0.540	0.511
2	0.01%	1.5	0.756	0.823
3	0.01%	2.5	1.26	1.61
4	0.01%	12	3.78	2.10
5	0.05%	0.5	0.756	2.54
6	0.05%	1.5	0.540	1.70
7	0.05%	2.5	3.78	2.63
8	0.05%	12	1.26	2.57
9	0.1%	0.5	1.26	1.02
10	0.1%	1.5	3.78	0.701
11	0.1%	2.5	0.540	1.72
12	0.1%	12	0.756	1.67
13	0.15%	0.5	3.78	0.603
14	0.15%	1.5	1.26	0.795
15	0.15%	2.5	0.756	1.41
16	0.15%	12	0.540	0.987

The results show that ECL intensity ranged from 0.511 V to 2.63 V under these different parameters. Table 2 lists the range analysis results of the orthogonal optimization. Here, k_i_ (i = 1, 2, 3, 4) is the average value of the four parallel results at the same level of the corresponding parameter, and r is the range. Table 2 shows that the relative importance of influence factors on the target index is as follows: the concentration of APTMS > the time of immersion (T) > the content of AuNPs. Thus, from these data, we decided that the optimal condition for electrode preparation is to drop 10 μL of 0.05% APTMS on the ITO for hydrolysis under 55 °C for 2.5 h and then array AuNPs on it with 50 μL of AuNPs sol.

**Table 2:** Range analysis of the L_16_(4^3^) orthogonal experiments.

No.	APTMs (V%)	T (h)	Au (m/10^−3^ g/mL)
k_1_	1.26	1.17	1.23
k_2_	2.36	1.00	1.61
k_3_	1.28	1.84	1.50
k_4_	0.948	1.83	1.51

**Figure 3:**
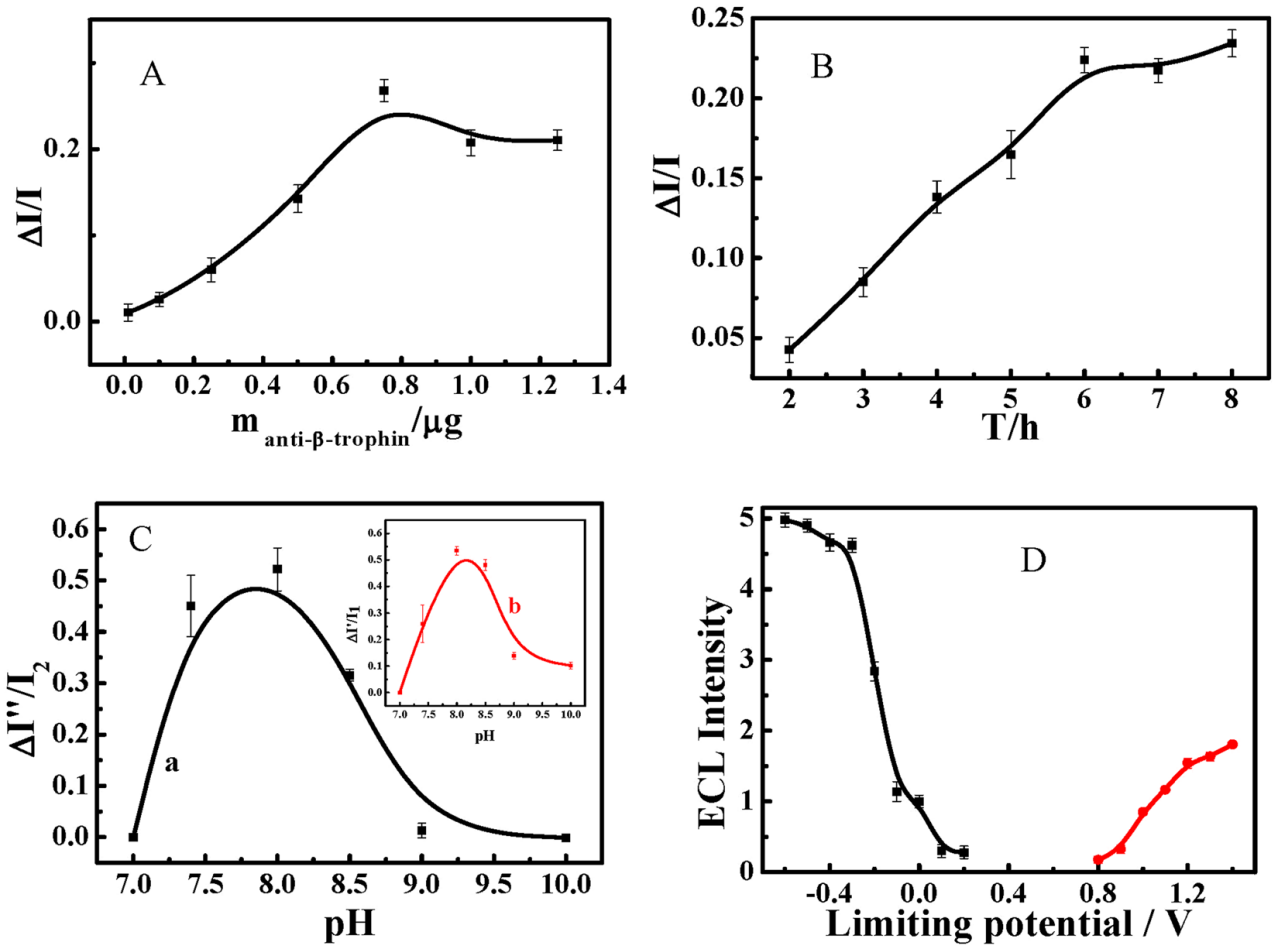
The optimization of (**A**) the effect of anti-β-trophin concentration; (**B**) The time of incubation; (**C**) The pH of PBS for (a) the detection of β-trophin and (b) the immobilization of anti-β-trophin; (**D**) The upper and lower limiting potential.

The immobilized quantity of anti-β-trophin on the electrode significantly affects the performance of the biosensor. As shown in Fig. 3A, the relative response (ΔECL/background) is greatest with a loading of 0.75 μg anti-β-trophin per piece of sensor. The incubation time is also important for fixing the antibody on the AuNPs-functionalized electrode; 6 h is necessary (Fig. 3B). The intensity of ECL emission towards β-trophin is related to the pH of the buffer solution. Figure 3C illustrates the ECL intensity of luminol in a 0.2 M buffer solution of pH ranging from 7.0 to 10.0. The relative intensity (ΔECL/background) is greatest at pH 8.0 when anti-β-trophin attaches to AuNPs/ITO, as displayed in the inset. The potential of the exciting electrolytic pulse also affects the ECL intensity. Figure 3D shows the increased ECL background accompanied by the decreasing lower limiting potential or increasing upper limiting potential. Considering the reversibility of the luminol redox reaction and ECL reproducibility, an upper limiting potential of 1.2 V and a lower limiting potential of −0.2 V were selected as the optimal conditions.

## Discussion

**Figure 4:**
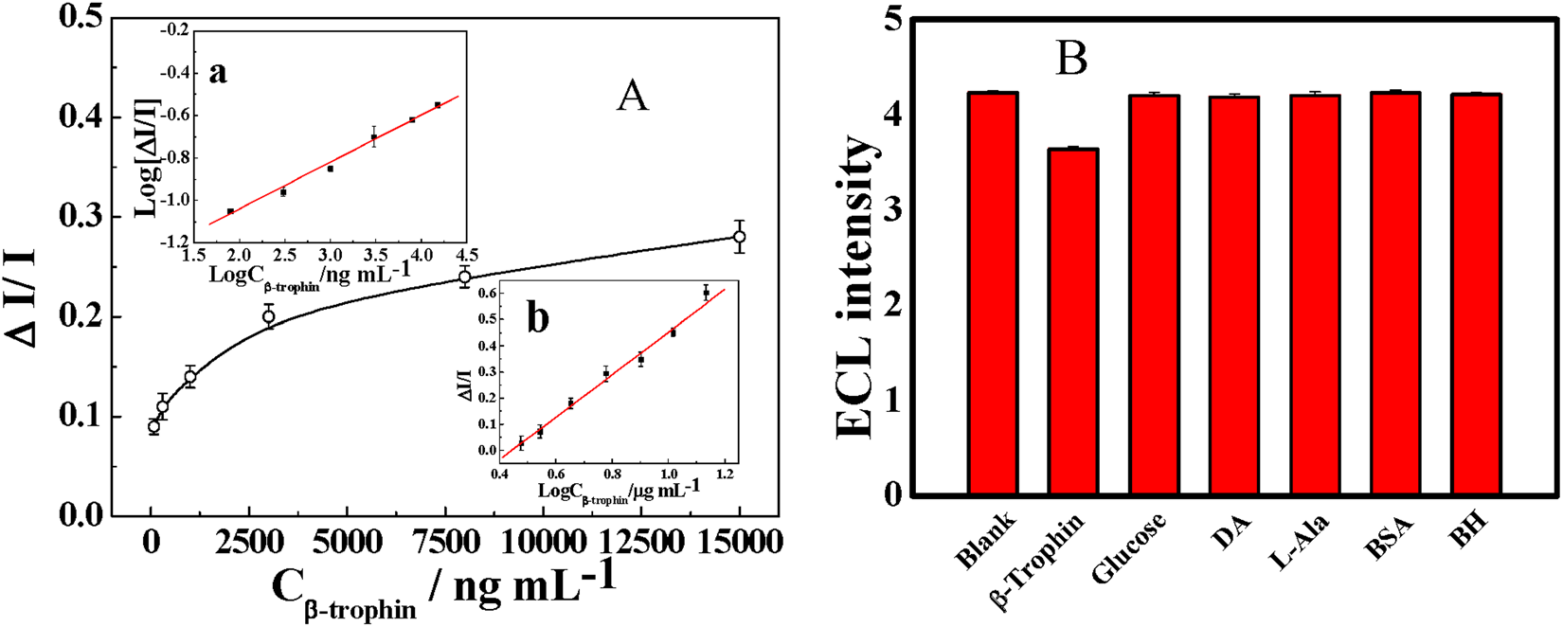
(**A**) The calibration of sensing output toward β-trophin. Experiment conditions: 10.0 μL of differently concentrated β-trophin (PBS, pH 7.4) drop on the sensor surface, incubated at 37 °C for 2 hours. (a) The linear calibration in a deoxygenated solution. (b) The linear calibration in dissolved oxygen equilibrium solution. (**B**) The interference of coexisting substances on the determination of β-trophin (300 ng mL^−1^).

Under the abovementioned optimal conditions, the ECL sensing capability for β-trophin of the resultant biosensor is shown in Fig. 4A. Here, the inserted figure “b” shows that the relative degree of quenched ECL (ΔI/I, ΔI as the decreased ECL signal after the antigen-antibody interaction) is linearly correlated with the logarithm of β-trophin concentration. A linear regression of the ECL signal against β-trophin concentration was established within the range of 3 μg mL^−1^ to 13.6 μg mL^−1^ with a detection limit (LOD) of 1.19 μg mL^−1^ (S/N = 3). The regression equation is ΔI/I = 0.81 log C_β-trophin_ − 0.36, with a correlation coefficient of 0.995.

However, this is a very poor sensitivity; as we known, generally the electrochemical immunosensors will extend their detection limit under the level of ng mL^−1^ even to pg mL^−1^ degree^34, 48^. On account of the dosage of 0.75 μg of β-trophin antibody on every piece of the sensor, and considering the effective contacting possibility of sensing interface for target proteins, we can estimate the top limit of the response. Conceivably, in this sensing system, the outputs are recorded after the completion of the immuno-reaction with enough time; thus, the rate of the immuno-reaction is non-essential. Here, a geometrical factor might be critical. It is expected that the proteins of the antibody were randomly orientated when they were attached to the AuNPs; thus, the antigenic determinant of the protein would be only partially functioning. Assuming a fully covered and ordered loading of antibody proteins on the sensing interface, at least 6.7% of total protein surface is motile for sensing event according to geometrical calculation (the effective percentage = 2πRh/4πR^2^, here the “R” is radius of globulin, “h” is the height of active section, for a 60° active section is (1 − √3/2)R). Thus, the expectable top limit should be at least 5.5 μg mL^−1^ (0.75 μg × 22 kDa/20 kDa × 1 mL/10 μL × 6.7%). Thereafter a ng mL^−1^ level detection limit is logical considering the millesimal resolution of our instrument.

Therefore, a giant gap between the measured result and the theoretical anticipation has appeared. What occurred in this situation? Are there any undiscovered tangles? Why?

**Figure 5:**
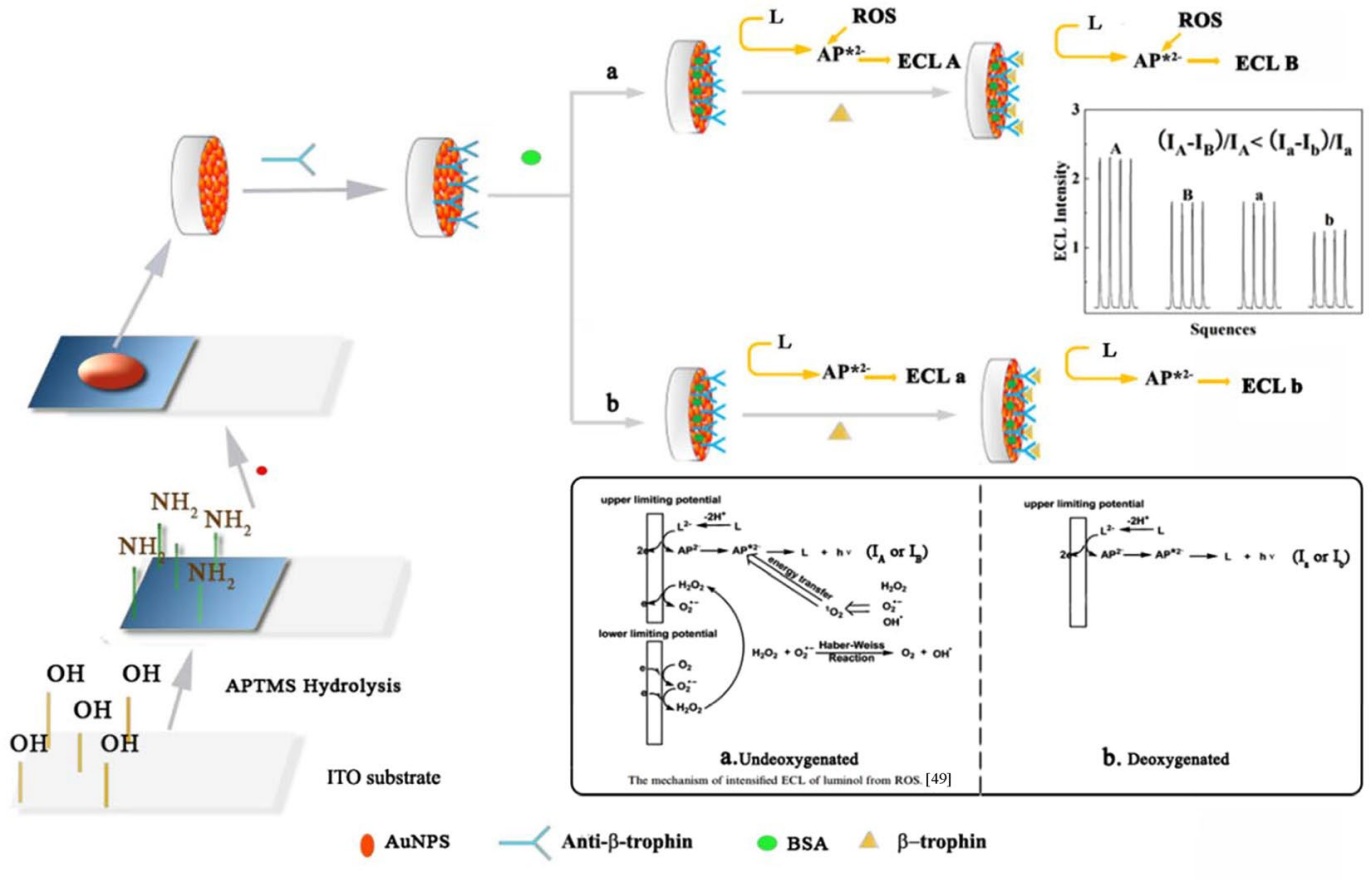
The sensor structure and response mechanism.

This tangle might be uncovered through careful consideration of the sensing mechanism. In this system, luminol serves as an ECL probe to monitor the immuno-interaction via the alteration of its mass transfer dynamics, which were controlled by the surface loading of the sensing interface. The ECL emission, occurring electrochemically on the surface of the electrode, depends on the mass transfer of the luminophore in a liquid medium and on a liquid/solid interface, is usually a sequential multiphase, multi-step process. In the present ECL immunosensor, luminol is obliged to pass through the solution by diffusion, then penetrate the gaps between the adhered antibody proteins to reach the surface of the AuNPs. When the β-trophin proteins are bonded on those immobilized antibodies, the mass transfer of luminol is more difficult owing to the complicated surface structure, which reduces the luminescent performance (as seen in Fig. 5). There are two main factors determining the mass-transfer behaviour of luminol: the occupied surface area and the thickness of the immobilized proteins. Both factors reduce the luminous efficiency of luminol. Therefore, the ECL output of luminol is contingent upon the status of the immuno-interaction on the sensor interface. Rationally, the sensing performance primarily relies on the speed and degree of the immuno-interaction.

But the above discussion cannot compensate the gap between theoretical and experimental data. To understand this case, another possible factor emerges as a clue. Indeed, there is a bypass reaction of oxygen that would influence the sensing performance^49, 50^. We can see from route “a” in Fig. 5 that the dissolved oxygen in solution will participate in the ECL reaction to boost the emission intensity with the forms of those reactive oxygen species (ROSs). Thus, there is a high ECL background that led to a low quenching ratio of immune-binding for the ECL of luminol. Therefore, a deoxygenated solution will be beneficial for sensing output (route “b”). As seen in Fig. 4A, there is a decayed exponential response of ΔI/I as a function of the concentration of β-trophin from ng mL^−1^ level to 15 μg mL^−1^. Furthermore, according to the decayed exponential fitting (the inset “a” in Fig. 4A), we can calibrate the response linearly against the concentration with a regression efficient of 0.996 (log ΔI/I = 0.22 log C_μ-trophin_ − 1.48) with a detection limit of 1.26 ng mL^−1^ and the quantification limit (LOQ) of 4.2 ng mL^−1^. Thus, the experimental results perfectly coincided with theoretical discussion, and our research successfully reached the desired object. Compared with those reported results of ELISA method, as listed in Table 3, the sensitivity of resultant immunosensor is rival.

**Table 3:** The sensing performance of some reported ELISA methods for β-trophin detection.

Sample	Lower limit of quantification (ng mL^−1^)	Country	Reference
Serum	~14.8	China	2
Serum	~22.8	Turkey	11
Serum	~12.3	Spain	51
Serum	~1.95	USA	52

The specificity of the resultant immunosensor was tested. Considering the possible coexistence of various substances in real samples as blood, the sensing responses of 300 ng mL^−1^ of β-trophin was examined together with some potential interferents including bovine hemoglobin (60 μg mL^−1^), L-alanine (150 μg mL^−1^), dopamine (150 μg mL^−1^), glucose (300 μg mL^−1^) and bovine serum albumin (300 μg mL^−1^). As shown in Fig. 4B, there is only 8.5% or less of ECL signal alteration from anyone of them, which demonstrates that this biosensor is hardly affected by those potential coexisting substances with the tolerance of at least 2000. The sensor has a good reproducibility, at 2.4% of RSD (n = 3) for 8.0 μg mL^−1^ β-trophin. The stability of the immunosensor was also examined by detections of 8.0 μg mL^−1^ β-trophin, and no evident alteration of intensity was observed during 28 days of storage at 4 °C. Thereafter, the resultant sensor was employed to detect β-trophin in inspissated serum samples. The results are shown in Table 4, it is clear that the detected results are closely accordance with ELISA. The results of recovery tests are listed in Table 5, between 80 to 90%, indicating a reliable quantification.

**Table 4:** The detected results of β-trophin in serum samples.

Sample No.	Background	Quenched by β-trophin	ΔI	ΔI/I	Quantified (ng mL^−1^)	ELISA (ng mL^−1^)
1	4.61	4.32	0.290	0.0629	1.43	1.46
2	4.83	4.51	0.320	0.0663	1.80	1.57
3	4.97	4.63	0.340	0.0684	2.07	2.09
4	4.77	4.44	0.330	0.0692	2.22	2.23

**Table 5:** The results of recovery tests.

β-Trophin content ng mL^−1^	β-Trophin added ng mL^−1^	Sensor background	Quenched by sample	ΔI/I	Found ng mL^−1^	Recovery %
1.43	100	4.84	4.42	0.087	81.3	79.9
	300	4.92	4.36	0.114	275.4	91.3
	800	4.97	4.27	0.141	724.4	90.4

## Methods

### Reagents and materials

ITO glass was purchased from Suzhou Nippon Sheet Glass Electronics Co. Ltd. (Suzhou, China). Luminol was obtained from Fluka (USA). β-Trophin (M_W_ = 22 kDa) and the antibody of β-trophin (anti-β-trophin, M_W_ = 20 kDa, 1 mg mL^−1^) were provided by Beijing Springup Scientific Co. Ltd. (Beijing, China). APTMS, chloroauric acid (HAuCl_4_·4H_2_O), trisodium citrate, bovine serum albumin (BSA, 96 to 99%) and phosphate (NaH_2_PO_4_·2H_2_O and Na_2_HPO_4_·12H_2_O) were obtained from Sinopharm Chemical Reagent Co. Ltd. (Shanghai, China). All chemicals and reagents are of analytical grade without further purification. A 0.2 M phosphate buffer solution (pH 8.0) containing 5 × 10^−8^ M luminol served as the electrolyte in ECL analysis. Ultrapure water is used throughout the experiments.

### Apparatus

ECL experiments were performed on a lab-built system as described in our previous paper^49^. An RST600 electrochemical workstation (custom-built, Risetest Instrument Co. Ltd., Suzhou, China) provided a pulsed electrolytic potential to excite and record the ECL signal. The ECL detector is an R212 photomultiplier tube (Hamamatsu, Japan) with a bias potential of −800 V. All experiments were performed in a 10-mL cuvette with a three-electrode system including a bare or modified ITO as the working electrode, a platinum wire as the auxiliary electrode and a silver wire as the reference electrode. Electrochemical studies were performed on an RST 5200 electrochemical workstation (Risetest, Suzhou, China). The form and size of the AuNPs were observed by transmission electron microscopy (FEI, USA). Scanning electron microscopy (S-4700, Hitachi, Japan) was applied to observe the surficial morphology of AuNPs/ITO and the resultant immunosensor.

### Preparation of biosensor

Prior to each experiment, a 1.0 cm × 5.0 cm ITO strip was cleaned in an ultrasonic bath with water, ethanol/1 M NaOH (1:1, v/v) and acetone successively, and then immersed in 30% (v/v) ammonium hydroxide for 12 h to acquire a hydrophilic surface with dense –OH groups. After nitrogen stream drying, the APTMS solution of anhydrous ethanol (0.05%, v/v) was dropped on and the full volatilization of ethanol was achieved, the obtained strip was laid in a wet environment at 55 °C to completely ensure the hydrolysis of APTMS.

AuNPs were synthesized according to our previous report^47^. Briefly, 4.5 mL of 1% sodium citrate solution was added to 100 mL of boiling 0.01% HAuCl_4_ solution to obtain AuNPs of approximately 15 nm. After 4 mL of freshly prepared AuNPs sol were twice centrifuged at 3000 rpm for 10 min and at 10000 rpm for 30 min, and finally dispersed with 250 μL of water, a homogeneously sized AuNPs sol was acquired (0.76 mg mL^−1^). Thereafter, some of the AuNPs sol (approximately 40 μg/ITO strip) was dropped onto the surface of APTMS/ITO to prepare a fully functionalized substrate. After that, the electrodes were rinsed using a copious amount of water and dried in a nitrogen stream.

### The fabrication of ECL immunosensor

The entire procedure of the fabrication of the immunosensor is clearly illustrated in Fig. 5. The antibody protein had compact affinity with AuNPs via an electrostatic interaction, so the consequential AuNPs/ITO electrode can provide a substrate for immobilizing the antibody with good bioactivity and stability. An aliquot of 10 μL of anti-β-trophin solution was dropped on an AuNPs/ITO electrode and incubated for 6 h at 25 °C, and then blocked by BSA solution (2%) for 2 h at 25 °C to avoid non-specific bindings. The resultant sensor was washed by PBS (pH 7.4) to remove those non-chemisorbed species after every step and stored at 4 °C.
